# Derivation of a clinical decision rule for termination of resuscitation in non-traumatic pediatric out-of-hospital cardiac arrest

**DOI:** 10.1016/j.resuscitation.2024.110400

**Published:** 2024-09-18

**Authors:** Pranav Shetty, Yunyi Ren, David Dillon, Alec Mcleod, Daniel Nishijima, Sandra L. Taylor, The CARES Surveillance Group

**Affiliations:** a Department of Emergency Medicine, University of California, Davis School of Medicine, 4150 V Street #2100, Sacramento, CA 95817, USA; b Department of Public Health Sciences, University of California, Davis, Medical Sciences 1-C, One Shield’s Ave. Davis, CA 95616, USA; c University of California, Davis School of Medicine, 4610 X St, Sacramento, CA 95817, USA

**Keywords:** Pediatric OHCA, Cardiac Arrest, Termination of Resuscitation, EMS Protocols, CARES

## Abstract

**Aim:**

Prehospital termination of resuscitation (ToR) rules are used to predict medical futility in adult out-of-hospital cardiac arrest (OHCA), however, the available evidence for pediatric patients is limited. The primary aim of this study is to derive a Pediatric Termination of Resuscitation (PToR) prediction rule for use in pediatric non-traumatic OHCA patients.

**Methods:**

We analyzed a retrospective cohort of pediatric OHCA patients within the CARES database over a 10-year period (2013–2022). We split the dataset into training and test datasets and fit logistic regressions with Least Absolute Shrinkage and Selection Operator (LASSO) to select predictor variables and estimate predictive test characteristics for the primary outcome of death and a secondary composite outcome of death or survival to hospital discharge with unfavorable neurologic status.

**Results:**

We analyzed a sample of 21,240 children where 2,326 (11.0%) survived to hospital discharge, and 1,894 (8.9%) survived to hospital discharge with favorable neurologic status. We derived a PToR rule for death demonstrating a specificity of 99.1% and a positive predictive value (PPV) of 99.8% and a PToR rule for death or survival with poor neurologic status with a specificity of 99.7% and PPV of 99.9% within the test dataset.

**Conclusion:**

We derived a clinical prediction rule with high specificity and positive predictive value in prehospital settings utilizing Advanced Life Support (ALS) providers which may inform termination of resuscitation considerations in pediatric patients. Further prospective and validation studies will be necessary to define the appropriateness and applicability of these PToR criteria for routine use.

## Introduction

Over 7,000 children experience an out of hospital cardiac arrest (OHCA) annually in the United States, which while less frequent than adults, similarly results in poor outcomes with only 11.3% surviving to hospital discharge, and 9.2% retaining good neurologic status.^1^ The majority of these events are attended by prehospital providers: first responders, emergency medical technicians (EMTs), and paramedics. When managing OHCA in adults, prehospital providers often utilize termination of resuscitation (ToR) rules to determine whether to terminate resuscitation in the field. ToR rules intend to reduce the number of futile cases transported through providing guidance on the predicted futility of continuing to treat a patient in cardiac arrest. Medical futility in this setting is commonly defined as a less than 1% chance of survival (also interpreted as a 99% positive predictive value (PPV) for death).^2^

The National Association of Emergency Medical Services Physicians (NAEMSP) recommends that all emergency medical services (EMS) providers have evidence-based protocols that allow for ToR in adults.^3^ ToR rules also contribute to public health by conserving the availability of scarce resources and decreasing the number of emergency vehicles using warning lights and sirens, which has shown to be associated with an increased likelihood of vehicle crashes.^4^ While ToR rules have proven effective for predicting medical futility in adults,^5–7^ studies applying those rules to pediatric patients have been limited, and children are often excluded from state, regional, and local EMS ToR protocols in the United States.^8^

Due to differences in the epidemiology and etiology of pediatric versus adult OHCA,^9^ as well as differing recommended resuscitation measures,^10^ further investigation is needed to derive and validate a ToR rule that can be appropriately applied to pediatric patients. Currently, the available evidence for pediatric prehospital ToR is extremely limited.^11^

The primary aim of this study is to derive a ToR clinical prediction rule for non-traumatic OHCA in patients under 18 years of age for use by advanced life support (ALS) EMS providers; a Pediatric Termination of Resuscitation (PToR) rule.

## Methods

### Study design

We sought to derive a PToR clinical prediction rule for use by ALS providers in patients under 18 years of age using the Cardiac Arrest Registry to Enhance Survival (CARES) database over a 10-year period (2013–2022). The CARES registry is a prospective, multicenter registry of patients with non-traumatic OHCA in the United States utilizing Utstein criteria for standard reporting, which as of 2022, covers over 50% of the US population.^12^ The test characteristics of the prediction rule (sensitivity, specificity, PPV, negative predictive value (NPV), positive likelihood ratio (LR+), and negative likelihood ratio (LR-)) were determined using those patients who did not survive to hospital discharge (primary outcome) and those patients who did not survive to hospital discharge or survived to hospital discharge with an unfavorable neurologic status (secondary outcome). The methodology of using non-survivors for the derivation and reporting of ToR rules, focusing on specificity and PPV, has been previously proposed as standard reporting in ToR studies.^13^ The UC Davis Institutional Review Board reviewed the research and determined it was not human subjects research and IRB review was not required (March 10th, 2022; Reference number: 1885169-1).

### Study population

We employed a retrospective observational cohort design for this study. Inclusion criteria included OHCA patients under the age of 18. Exclusion criteria included cases for which no outcome data was available. For the sensitivity analysis, patients who were pronounced dead in the field were excluded.

### Outcome measures

The primary outcome was non-survival to hospital discharge; this included death in the field, death prior to hospital admission, or death during hospitalization. The secondary outcome was non-survival to hospital discharge or survival to hospital discharge with unfavorable neurologic status. The Pediatric Cerebral Performance Category Scale (PCPC) was used to determine the neurologic status of survivors in this study. The PCPC scale is scored from 1 to 6 where 1 = Normal; 2 = Mild disability; 3 = Moderate disability; 4 = Severe disability; 5 = Coma or vegetative state; and 6 = Brain death.^14^ Reporting of unfavorable outcomes are varied in the literature^15^ and most commonly use a PCPC of either 3 – 6 or 4 – 6 to reflect unfavorable neurologic status. The CARES database only reports Adult CPC scores which are graded 1 – 5 where a PCPC score of 2 or 3 is reported as a CPC score of 2. In our study we considered unfavorable neurological status to include patients with a CPC score of ≥ 3 which equates to a PCPC score of ≥ 4.

### Predictive factors

Predictive variables evaluated were Arrest Witnessed Status, Bystander CPR, Rhythm, Etiology of Arrest, and Sustained ROSC; Sustained ROSC is defined as ROSC for 20 consecutive minutes or a pulse present at the end of EMS care regardless of duration. These variables were chosen as they are consistent with those utilized in adult ToR rules, as well as those which have been previously utilized in pediatric patients.^16,17^ “Arrest Witnessed Status” was categorized as witnessed/unwitnessed. “Bystander CPR” was categorized as yes/no. “Rhythm” was created from the original variable “First Monitored Rhythm” to classify cardiac rhythm as shockable (ventricular tachycardia, ventricular fibrillation, and unknown shockable), non-shockable (idioventricular/pulseless electrical activity (PEA) and unknown unshockable), or asystole. Asystole was examined separately from other non-shockable cardiac rhythms as previous studies have shown the association of worse outcomes with asystole as the presenting rhythm.^18^ We dichotomized “Etiology of Arrest” into drowning/electrocution versus all other presumed causes. Drowning/electrocution was examined separately as previous research suggests that these causes of cardiac arrest may be associated with improved survival.^19,20^ We considered including a variable for whether the patient was defibrillated, but this variable was highly correlated with “Rhythm” (Cramer V = 0.6725); additionally, a large proportion of patients noted to have shockable rhythms were defibrillated (98.2%).

### Statistical analysis

We split the full dataset into training (2013–2019) and test (2020–2022) datasets. We specifically selected the test set to encompass the period 2020–2022 during the peak of the COVID-19 pandemic to a) use non-pandemic period data for model development and b) assess model performance on data with potentially different characteristics than the training set. Using the training dataset, we fit logistic regressions with Least Absolute Shrinkage and Selection Operator (LASSO) to select predictors and estimate model coefficients. The penalty parameter (lambda) was estimated using ten-fold cross validation with the objective of maximizing the area under the receiver operating characteristic curve (AUROC). Using training set results, we identified a predicted probability for our decision rule threshold to provide a specificity of at least 99%. Models developed with the training set were then evaluated on the test set. We report AUROC, sensitivity, specificity, PPV, NPV, LR+, and LR− estimates with 95% confidence intervals for each model and dataset.

For ideal derivation of ToR criteria, all OHCA patients are transported to the hospital; however, this is not standard practice in the US healthcare system. In order to address this limitation, a sensitivity analysis was conducted to examine only the subset of patients who were transported to the hospital, excluding those patients for whom resuscitative efforts were terminated in the field per local protocol; this subset comprised 86% of the total study population. Details regarding decisions to terminate resuscitation in the field for specific cases were not available within the dataset. All analyses were conducted using R Statistical Software Version 4.1.3.

### Model bias assessment

Racial bias in clinical prediction models have shown the potential for perpetuating or exacerbating existing health disparities.^21^ Therefore, we evaluated model performance by race/ethnicity and gender groups. Race/Ethnicity was assigned by the EMS provider as reported by the patient, family, or healthcare provider and was analyzed as White, Black, Asian, Native American, Hispanic (regardless of race), and Unknown/Mixed. Gender was assigned by the EMS provider as determined by the provider or by self-report and was analyzed as Female and Male; there were two transgender children in the dataset that were dropped for the purpose of these analyses given such a small sub-group would be susceptible to overgeneralization of results.

## Results

**Fig. 1 –:**
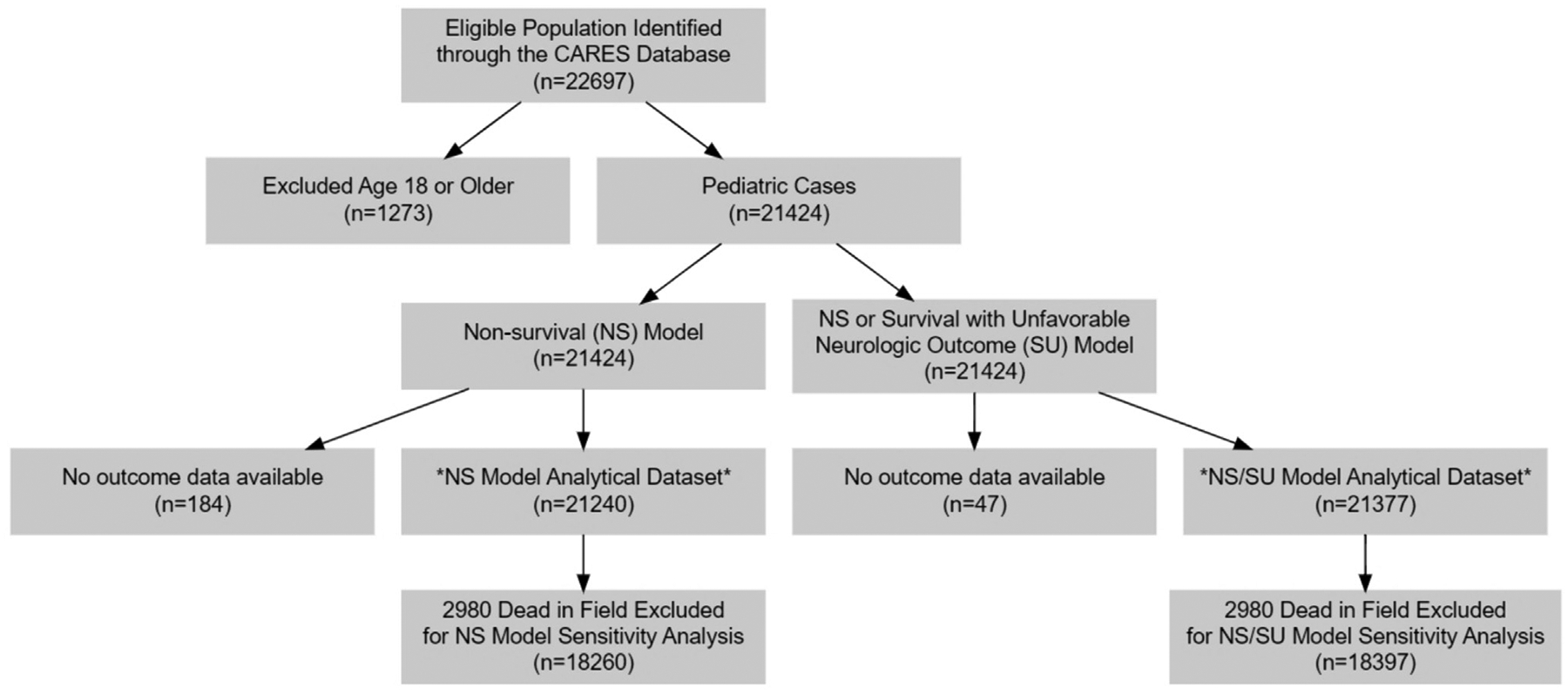
Study Flow Diagram.

The full dataset included 22,697 children. We first conducted a complete case analysis, restricting the analytical dataset to the study sample of 21,240 children for the primary outcome and 21,377 for the secondary outcome after application of inclusion and exclusion criteria. Fig. 1 shows the study flow diagram. Mean (S.D.) age was 4.3 (5.8) years and 41.4% were female. 5,721 (26.9%) patients survived to hospital admission, 2,326 (11.0%) survived to hospital discharge, and 1,894 (8.9%) survived to hospital discharge with favorable neurologic outcome. Table 1 shows overall patient characteristics disaggregated by clinical outcome. We then conducted a sensitivity analysis excluding children for whom resuscitative efforts were terminated in the field; 18,260 children were included for the primary outcome and 18,397 for the secondary outcome.

### Primary outcome: Non-survival to hospital discharge

**Fig. A1 –:**
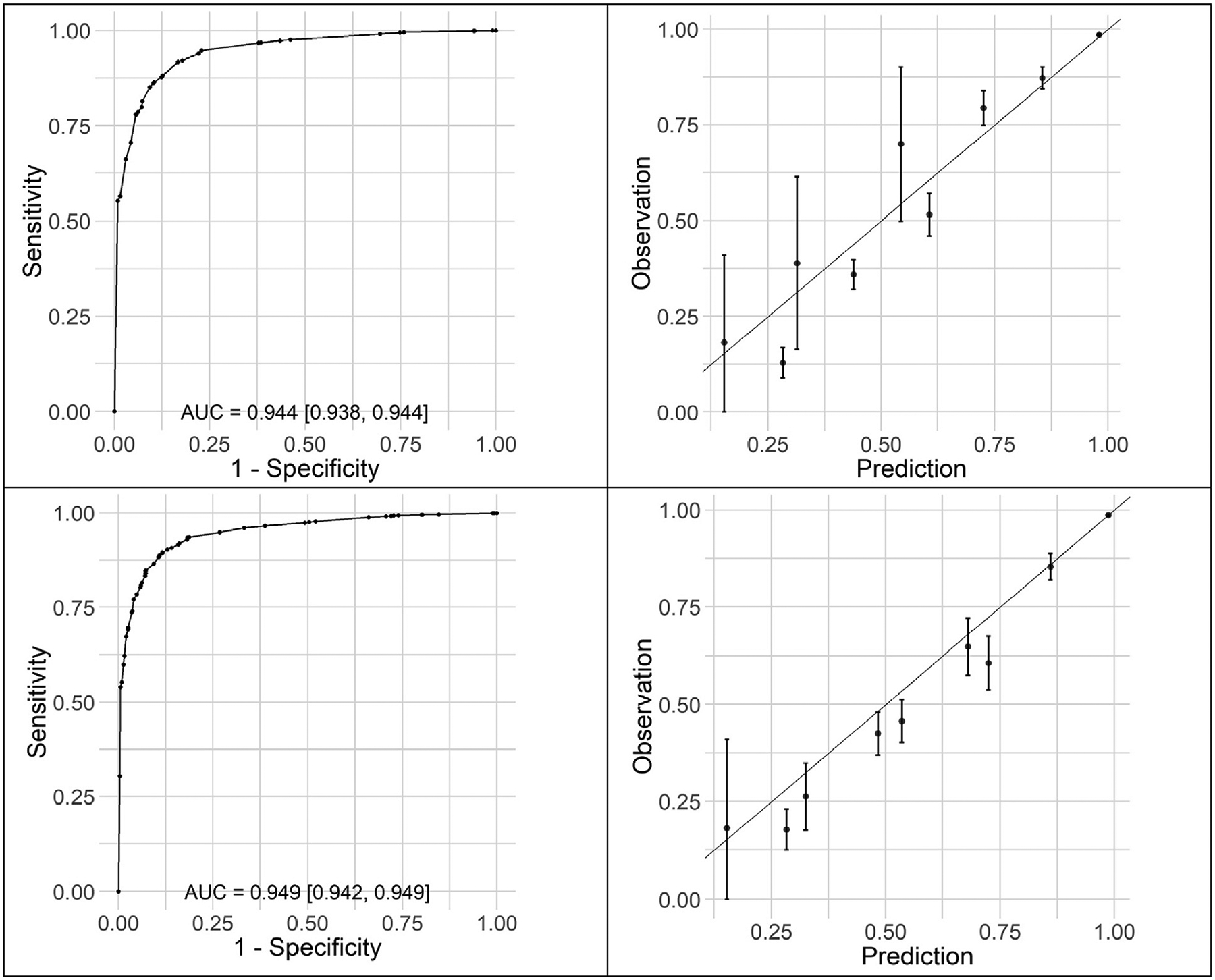
Figures of Receive Operating Characteristic (ROC) and calibration curves for each model applied to the test set. (1) ROC Curve for Model for Non-survival to hospital discharge– Upper Left. (2) Calibration Curve for Model for Non-survival to hospital discharge - Upper Right. (3) ROC Curve for Model for Non-survival to hospital discharge or survival with unfavorable neurologic status – Lower Left. (4) Calibration Curve for Model for Non-survival to hospital discharge or survival with unfavorable neurologic status – Lower Right.

Using the training dataset, unwitnessed arrest, initial absence of cardiac electrical activity (asystole), arrest not due to drowning or electrocution, and absence of sustained ROSC were all associated with an increased likelihood of non-survival to hospital discharge (Table 2). Bystander CPR was not retained in the model. We selected a predicted probability of 98.37% as the decision threshold for predicting survival or non-survival; this corresponded to a specificity of 97.83% (the highest value less than 100%) and a PPV [95% confidence interval] of 99.42% [99.19%, 99.60%]. Similar results were obtained when applied to the test set, including a specificity of 99.14% [98.37, 99.60] and a PPV of 99.8% [99.63, 99.91]. Full performance metrics for the model are provided in Table 3. The AUROC of the model applied to the test set was 0.944 [0.938, 0.951] and was well calibrated (Appendix Figure A1).

The sensitivity analysis identified the same factors as being associated with an increased likelihood of non-survival to hospital discharge. Within the training dataset we found a specificity of 97.83% and PPV of 99.42% [99.13, 99.64]. For the test dataset we obtained similar results including a specificity of 99.14% [98.37, 99.60] and a PPV of 99.75% [99.52, 99.88].

### Secondary outcome: Non-survival to hospital discharge or survival to hospital discharge with unfavorable neurologic status

In the training dataset, unwitnessed arrest, no bystander CPR, initial absence of cardiac electrical activity (asystole), arrest not due to drowning or electrocution, and absence of sustained ROSC were retained in the model, and all were associated with an increased likelihood of non-survival to hospital discharge or survival to hospital discharge with unfavorable neurologic status (Table 2). We selected a predicted probability of 99.65% as the decision threshold for predicting non-survival to hospital discharge or survival with unfavorable neurological status versus survival with good neurologic status; this corresponded to a specificity of 99.1% and a PPV of 99.72% [99.47, 99.87]. When applied to the test set, we obtained a specificity of 99.66% [99.01, 99.93] and a PPV of 99.88% [99.66, 99.98]. Full performance metrics for the model are provided in Table 3. The AUROC of the model applied to the test set was 0.949 [0.942, 0.955] and was well calibrated (Appendix Figure A1).

The sensitivity analysis identified the same factors associated with an increased likelihood of non-survival to hospital discharge or survival with unfavorable neurologic outcome with the exception that bystander CPR was not retained in the model. Within the training dataset we found a specificity of 99.0% and a PPV of 99.79% [99.59, 99.91]. For the test dataset we obtained similar results including a specificity of 99.43% [98.69, 99.82] and a PPV of 99.86% [99.67, 99.95].

We conducted a gender and race/ethnicity assessment to evaluate performance across reported gender and race/ethnicity for both outcome variables (Appendix Table A1 and A2). Within this assessment no meaningful differences regarding model performance between gender and race/ethnicity groups were found.

To evaluate the predictive value of the PToR criteria across different age groups, an age stratification assessment was performed, showing that the criteria had similar model performance in infants (<1 year old), children (1–11 years old), and adolescents (12 or older). These specific age categories have been used in previous studies evaluating pediatric OHCA within the CARES registry.^22^ For the primary outcome, the PPV in the infant group was 0.998 [0.996, 0.999], compared to a PPV of 0.997 [0.99, 0.999] in the children group, and a PPV of 1 [0.995, 1] in the adolescent group. For the secondary outcome, the PPV in the infant group was 0.998 [0.995, 1], compared to a PPV of 1 [0.993, 1] in the children group, and a PPV of 1 [0.99, 1] in the adolescent group. (Appendix Table A3).

## Discussion

We derived a clinical prediction rule with high specificity and positive predictive value to inform termination of resuscitation considerations in pediatric OHCA patients in the prehospital setting. The PToR clinical decision rule for non-survival to hospital discharge consists of four criteria: 1) Unwitnessed arrest; 2) Absence of cardiac electrical activity (asystole); 3) Arrest not due to drowning or electrocution; and 4) No sustained ROSC. When all the PToR criteria are present, the rule demonstrated a specificity of 99.1% and PPV of 99.8% for non-survival to hospital discharge within the test set. The PToR clinical decision rule for non-survival to hospital discharge or survival with unfavorable neurologic status consists of five criteria: 1) Unwitnessed arrest; 2) Absence of cardiac electrical activity (asystole); 3) Arrest not due to drowning or electrocution; 4) No sustained ROSC; and 5) No bystander CPR. When all PToR criteria are present, the rule demonstrated a specificity of 99.1% and PPV of 99.8% for non-survival to hospital discharge or survival with unfavorable neurologic status within the test set.

Significant variation in the implementation of pediatric OHCA protocols within US EMS systems exists; over 38% of protocols do not specify even when to initiate transport after arrest.^23^ Some of this variability is likely due to the overall lack of evidence regarding pediatric OHCA and if, or when, ToR in the field is warranted.^24^ A Maryland study independently developed PToR criteria and demonstrated a PPV of 97.8% for return of spontaneous circulation (ROSC), but did not include outcomes after hospital arrival.^25^ Rotering et al., found that applying current BLS adult ToR rules to pediatric patients yielded a PPV of 93.3%, lower than the commonly accepted threshold for medical futility.^26^

Additionally, the heightened emotions EMS providers experience when encountering a pediatric cardiac arrest,^27^ the lack of comfort in withholding resuscitation in this population,^28^ and the presence of “non-medical” factors such as fear of liability, provider experience, or their moral/ethical perspective^29^ also likely contribute to lack of standardization in practice. Prehospital providers may not feel comfortable discussing ToR for pediatric patients with family members,^30^ so a systematic approach involving parents in decision-making is important for emotional and ethical reasons.

The criteria derived in our study are similar to those used for adult prehospital ToR with most adult ToR rules assessing the factors of 1) Unwitnessed Arrest; 2) Shock delivery; 3) Presence of ROSC; and 4) Bystander CPR.^31^ Given we had two different outcome measures which are both generally accepted to represent poor patient outcomes (non-survival to hospital discharge and non-survival to hospital discharge or survival with unfavorable neurologic status), we derived two different PToR rules. The rules were very similar except that the rule for non-survival to hospital discharge or survival with unfavorable neurologic status included assessment of bystander CPR. The choice of which PToR rule to use will depend on the outcome the user is attempting to predict (non-survival to hospital discharge alone versus non-survival to hospital discharge or survival with poor neurologic status). As our study conducted a primary derivation of PToR criteria, the findings may be more generalizable to the pediatric population as compared to prior studies which retrospectively applied adult ToR criteria to pediatric patients. Additionally, these PToR criteria may be utilized in future research studies by better defining the sub-group of non-futile cases who may be more likely to benefit from novel therapeutic interventions.

We elected to include the presumed etiology of arrest within our model as a possible predictor. Previous studies have shown that initial determination of the etiology of arrest, particularly in the absence of ventricular fibrillation as the presenting rhythm where inherent cardiac causes are likely, can be inaccurate.^32,33^ However, determining whether drowning or electrocution contributed to arrest is likely more precise given the environmental elements present at the scene and bystander reports. It has been shown that both drowning^34^ and electrocution^35^ may result in improved patient outcomes after cardiac arrest and this was found in our study as well; as such cardiac arrest due to drowning or electrocution would exclude a pediatric patient from prehospital ToR.

Our study had several limitations. We utilized a prospective EMS registry (CARES); however, we conducted a retrospective analysis of this data which was limited to information available in the dataset. For example, the available information on ROSC described “Sustained ROSC” which was present if the patient had ROSC for 20 consecutive minutes during prehospital care or had a pulse on arrival to the ED (even if less than 20 min).^36^ Most adult ToR rules include any ROSC,^37^ irrespective of duration, as an exclusion to ToR which makes comparisons between the PToR and adult ToR criteria challenging. As EMS providers would generally not wait for sustained ROSC prior to transport, a proxy of any ROSC may be a more suitable criterion and should be studied further. A second limitation is that some patients (14%) had what was likely ascertained as a low likelihood of survival, either through jurisdiction-specific EMS protocols, or a decision made by EMS medical control, and their resuscitation was terminated in the field. In the ideal derivation of a ToR protocol, all patients are transported to determine final outcomes through full provision of care. We attempted to address this limitation through the sensitivity analysis which provided generally the same criteria as being associated with the outcomes of interest and with equivalent specificity and PPV. Third, our data does not consider variability in clinical care provided to any patient, both within the prehospital or in-hospital setting, which is known to impact survival rates.^38^ We selected the test dataset to occur during the peak of the COVID-19 pandemic, which had the methodologic benefit of test data with potentially different characteristics than the training data, but may represent a time period where variable approaches to OHCA resuscitation occurred.

## Conclusions

Our study derived prehospital PToR criteria with high specificity and positive predictive values for poor pediatric outcomes after OHCA. Medical providers not utilizing ToR criteria exhibit significant variability in their decision to terminate resuscitation in the prehospital setting.^39^ The ideal ToR criteria would have a near-perfect ability to predict death, thus being able to discriminate between patients in whom further resuscitation is warranted versus those in which it would be futile. ToR protocols may reduce demands on the emergency healthcare system and allow for use of limited resources for individuals who are most likely to benefit.^40^ Further prospective and validation studies are necessary to define the appropriateness and applicability of these PToR criteria for use in the prehospital setting.

## Figures and Tables

**Table 1 –:** Patient characteristics by clinical outcome.

	Total (N = 21377)	Non-survival to hospital discharge (N = 19075)	Survival with unfavorable neurologic status (N = 408)	Survival with favorable neurologic status (N = 1894)
**Age**				
Mean (SD)	4.3 (5.8)	4.0 (5.6)	5.0 (5.4)	7.1 (6.5)
Median	0.9	0.8	2	5
Q1, Q3	0.2, 8.0	0.2, 7.0	0.8, 9.0	0.9, 14.0
Range	0.0–17.0	0.0–17.0	0.0–17.0	0.0–17.0
N-Miss	0	0	0	0
**Gender**				
Female	8847 (41.4%)	8044 (42.2%)	140 (34.3%)	663 (35.0%)
Transgender	2 (0.0%)	1 (0.0%)	0 (0.0%)	1 (0.1%)
Male	12,528 (58.6%)	11,030 (57.8%)	268 (65.7%)	1230 (64.9%)
**Race/Ethnicity**				
Asian	411 (1.9%)	357 (1.9%)	8 (2.0%)	46 (2.4%)
Black	6941 (32.5%)	6354 (33.3%)	129 (31.6%)	458 (24.2%)
Hispanic	2589 (12.1%)	2283 (12.0%)	58 (14.2%)	248 (13.1%)
Native American	142 (0.7%)	130 (0.7%)	2 (0.5%)	10 (0.5%)
Pacific Islander	139 (0.7%)	128 (0.7%)	1 (0.2%)	10 (0.5%)
Unknown	4269 (20.0%)	3794 (19.9%)	89 (21.8%)	386 (20.4%)
White	6886 (32.2%)	6029 (31.6%)	121 (29.7%)	736 (38.9%)
**Arrest Witnessed Status**				
Unwitnessed	15,357 (71.8%)	14,589 (76.5%)	198 (48.5%)	570 (30.1%)
Witnessed by 911 Responder	966 (4.5%)	748 (3.9%)	44 (10.8%)	174 (9.2%)
Witnessed by Bystander	5054 (23.6%)	3738 (19.6%)	166 (40.7%)	1150 (60.7%)
**Bystander CPR?**				
No	10,302 (48.2%)	9530 (50.0%)	163 (40.0%)	609 (32.2%)
Not Applicable	1316 (6.2%)	1029 (5.4%)	65 (15.9%)	222 (11.7%)
Yes	9759 (45.7%)	8516 (44.6%)	180 (44.1%)	1063 (56.1%)
**Rhythm**				
Asystole	15,058 (70.4%)	14,658 (76.8%)	173 (42.4%)	227 (12.0%)
Non-shockable^1^	4860 (22.7%)	3571 (18.7%)	198 (48.5%)	1091 (57.6%)
Shockable	1459 (6.8%)	846 (4.4%)	37 (9.1%)	576 (30.4%)
**Etiology**				
Drowning/Electrocution	1900 (8.9%)	1484 (7.8%)	84 (20.6%)	332 (17.5%)
Other Etiologies^2^	19,477 (91.1%)	17,591 (92.2%)	324 (79.4%)	1562 (82.5%)
**Sustained ROSC - Yes/No**				
No	17,025 (79.6%)	16,713 (87.6%)	126 (30.9%)	186 (9.8%)
Yes	4352 (20.4%)	2362 (12.4%)	282 (69.1%)	1708 (90.2%)

1 Includes idioventricular/pulseless electrical activity (PEA) and unknown unshockable rhythm.

2 Includes presumed cardiac, respiratory/asphyxia, exsanguination/hemorrhage, drug overdose, and other.

**Table 2 –:** Point estimates for independent variables from LASSO logistic regression models predicting non-survival to hospital discharge and non-survival to hospital discharge or survival with unfavorable neurologic status. Values of 0 indicate the variable was dropped from model.

		Non-survival to hospital discharge	Non-survival to hospital discharge or survival with unfavorable neurologic status
Predictors	Label	Estimate	Estimate
**(Intercept)**		4.571	5.758
**Arrest Witnessed Status**	Witnessed by 911 Responder	−0.569	−0.799
	Witnessed by Bystander	−0.687	−0.805
	Unwitnessed	Ref	Ref
**Bystander CPR?**	Yes	0	−0.201
	Not Applicable	0	0
	No	Ref	Ref
**Rhythm**	Shockable	−1.875	−2.572
	Non-shockable	−1.229	−1.688
	Asystole	Ref	Ref
**Etiology**	Drowning/Electrocution	−0.800	−0.869
	Other Etiologies	Ref	Ref
**Sustained ROSC**	Yes	−2.909	−3.113
	No	Ref	Ref

**Table 3 –:** Performance metrics for the LASSO logistic regression models predicting non-survival to hospital discharge and non-survival to hospital discharge or survival with unfavorable neurologic outcome by different subsets of data.

Model	Set	Specificity	Sensitivity	PPV	NPV	^3^LR+	^4^LR−
***Non-survival to hospital discharge***	**Train**	97.83%^1^	55.3% [54.07, 55.98]	99.42% [99.19%, 99.60%]	20.75% [19.73, 21.80]	25.49 [24.89, 26.11]	0.46 [0.45, 0.47]
	**Test**	99.14% [98.37, 99.60]	55.18% [54.10, 56.25]	99.8% [99.63, 99.91]	17.18% [16.23, 18.16]	64.13 [63.23, 65.04]	0.45 [0.44, 0.46]
	**Train – EDF** ^2^	97.83%^1^	52.65% [51.49, 53.81]	99.42% [99.13, 99.64]	22.54% [21.31, 23.8]	24.28 [23.72, 24.85]	0.48 [0.47, 0.49]
	**Test – EDF**	99.14% [98.37, 99.60]	51.46% [50.27 52.65]	99.75% [99.52, 99.88]	23.45% [22.2, 24.73]	59.84 [59.06, 60.64]	0.49 [0.48, 0.50]
***Non-survival to hospital discharge or survival with unfavorable neurologic status***	**Train**	99.1%^1^	29.58% [28.73, 30.45]	99.72% [99.47, 99.87]	11.47% [10.81, 12.16]	32.87 [32.33, 33.42]	0.71 [0.70, 0.72]
	**Test**	99.66% [99.01, 99.93]	30.43% [29.46, 31.42]	99.88% [99.66, 99.98]	10.14% [9.51, 10.79]	89.56 [88.22, 90.92]	0.70 [0.69, 0.71]
	**Train – EDF**	99.0%^1^	51.19% [50.05, 52.33]	99.79% [99.59, 99.91]	18.0% [16.88, 19.16]	51.19 [50.37, 52.02]	0.49 [0.48, 0.50]
	**Test – EDF**	99.43% [98.69, 99.82]	50.16% [48.99, 51.33]	99.86% [99.67, 99.95]	19.79% [18.63, 20.99]	88.46 [87.14, 89.79]	0.50 [0.49, 0.51]

1 No confidence interval provided for training set specificity because we chose the model threshold based on the highest specificity.

2 EDF = the subset of data excluding patients that died in the field.

3 Positive Likelihood Ratio.

4 Negative Likelihood Ratio.

## Appendix

**Appendix Table A1 –:** Performance metrics by race and gender for the model evaluating non-survival to hospital discharge applied to the test set.

	AUROC	Sensitivity	Specificity	PPV	NPV	LR+	LR-
**Native American^1^**	0.971 [0.929,1]	0.542 [0.392,0.686]	1 [0.398,1]	1 [0.868,1]	0.154 [0.044,0.349]	542.00 [237.74, 1140.45]	0.46 [0.31, 0.61]
**Asian**	0.947 [0.907,0.988]	0.5 [0.424,0.576]	1 [0.872,1]	1 [0.959,1]	0.233 [0.159,0.32]	500.00 [331.64, 573.39]	0.50 [0.42, 0.58]
**Black^2^**	0.952 [0.939,0.964]	0.585 [0.566,0.603]	0.988 [0.965,0.998]	0.998 [0.995,1]	0.175 [0.156,0.196]	48.75 [46.20, 51.55]	0.42 [0.40, 0.44]
**Hispanic^3^**	0.953 [0.94,0.967]	0.529 [0.499,0.559]	1 [0.979,1]	1 [0.994,1]	0.247 [0.215,0.281]	529.00 [488.86, 571.23]	0.47 [0.44, 0.50]
**Pacific Islander^4^**	0.99 [0.967,1]	0.509 [0.373,0.644]	1 [0.541,1]	1 [0.881,1]	0.176 [0.068,0.345]	509.00 [259.37, 629.79]	0.49 [0.36, 0.63]
**Unknown^5^**	0.938 [0.921,0.956]	0.553 [0.527,0.579]	0.989 [0.96,0.999]	0.997 [0.991,1]	0.213 [0.186,0.243]	50.27 [47.98, 52.63]	0.45 [0.42, 0.48]
**White**	0.935 [0.923,0.947]	0.531 [0.512,0.55]	0.99 [0.975,0.997]	0.997 [0.993,0.999]	0.244 [0.223,0.265]	53.10 [50.83, 55.44]	0.47 [0.45, 0.49]
**Female**	0.945 [0.935,0.956]	0.557 [0.54,0.573]	0.994 [0.98,0.999]	0.999 [0.996,1]	0.183 [0.166,0.201]	92.83 [88.63, 97.18]	0.44 [0.42, 0.46]
**Male**	0.944 [0.935,0.952]	0.548 [0.534,0.562]	0.99 [0.979,0.996]	0.997 [0.995,0.999]	0.24 [0.225,0.256]	54.80 [53.35, 56.29]	0.46 [0.44, 0.48]

**Appendix Table A2 –:** Performance metrics by race and gender from the model for non-survival to hospital discharge or survival with unfavorable neurologic status model applied to the test set.

	AUROC	Sensitivity	Specificity	PPV	NPV	LR+	LR-
**Native American** ^1^	0.98 [0.945,1]	0.32 [0.195,0.467]	1 [0.292,1]	1 [0.794,1]	0.081 [0.017,0.219]	Inf [2.17, Inf]	0.68 [0.53, 0.81]
**Asian**	0.939 [0.895,0.983]	0.254 [0.93,0.323]	1 [0.863,1]	1 [0.925,1]	0.153 [0.102,0.218]	Inf [6.38, Inf]	0.75 [0.68, 0.82]
**Black** ^2^	0.958 [0.946,0.97]	0.35 [0.333,0.368]	0.995 [0.973,1]	0.999 [0.994,1]	0.098 [0.086,0.112]	70.00 [50.69, 119.36]	0.65 [0.63, 0.67]
**Hispanic/Latino** ^3^	0.95 [0.935,0.965]	0.339 [0.312,0.368]	1 [0.975,1]	1 [0.99,1]	0.164 [0.141,0.19]	Inf [12.48, Inf]	0.66 [0.63, 0.69]
**Pacific Islander** ^4^	0.991 [0.972,1]	0.276 [0.167,0.409]	1 [0.541,1]	1 [0.794,1]	0.125 [0.047,0.252]	Inf [2.19, Inf]	0.72 [0.59, 0.83]
**Unknown/Mixed** ^5^	0.945 [0.929,0.96]	0.287 [0.264,0.31]	1 [0.975,1]	1 [0.991,1]	0.121 [0.103,0.14]	Inf [11.50, Inf]	0.71 [0.68, 0.74]
**White**	0.941 [0.929,0.952]	0.256 [0.24,0.273]	0.994 [0.98,0.999]	0.997 [0.99,1]	0.121 [0.103,0.14]	42.67 [27.66, 56.25]	0.75 [0.72, 0.77]
**Female**	0.95 [0.939,0.96]	0.31 [0.295,0.326]	0.997 [0.981,1]	0.999 [0.995,1]	0.104 [0.093,0.116]	103.33 [61.50, 137.69]	0.69 [0.67, 0.71]
**Male**	0.948 [0.94,0.956]	0.3 [0.287,0.313]	0.997 [0.988,1]	0.999 [0.995,1]	0.147 [0.136,0.158]	90.00 [65.45, 106.61]	0.70 [0.68, 0.72]

1 Native American = American-Indian/Alaska Native.

2 Black = Black/African-American.

3 Hispanic/Latino = Black/African-American,Hispanic/Latino; Hispanic/Latino; White,Hispanic/Latino.

4 Pacific Islander = Native Hawaiian/Pacific Islander.

5 Unknown/Mixed = Unknown; American-Indian/Alaska Native,White; Asian,Black/African-American; White,Black/African-American; Native Hawaiian/Pacific Islander,Asian; Native Hawaiian/Pacific Islander,White,Black/African-American; White,Black/African-American.

**Appendix Table A3 –:** Performance metrics by age stratification^1^ from the model for non-survival to hospital discharge and non-survival to hospital discharge or survival with unfavorable neurologic status model applied to the test set.

	Non-survival to hospital discharge			Non-survival to hospital discharge or survival with unfavorable neurologic status		
Age Group	< 1 Yrs	1 – 11 Yrs	≥12 Yrs	< 1 Yrs	1 – 11 Yrs	≥12 Yrs
**AUC**	0.951 [0.937,0.964]	0.921 [0.907,0.934]	0.942 [0.931,0.953]	0.955 [0.940,0.969]	0.927 [0.914,0.94]	0.945 [0.935,0.956]
**Sens**	0.703 [0.689,0.717]	0.371 [0.352,0.391]	0.441 [0.417,0.465]	0.398 [0.383,0.413]	0.203 [0.187,0.219]	0.226 [0.206,0.246]
**Spec**	0.977 [0.95,0.991]	0.993 [0.98,0.999]	1 [0.99,1]	0.986 [0.961,0.997]	1 [0.989,1]	1 [0.989,1]
**PPV**	0.998 [0.996,0.999]	0.997 [0.99,0.999]	1 [0.995,1]	0.998 [0.995,1]	1 [0.993,1]	1 [0.99,1]
**NPV**	0.17 [0.151,0.19]	0.217 [0.199,0.235]	0.274 [0.249,0.299]	0.143 [0.129,0.157]	0.143 [0.129,0.157]	
**LR+**	30.57 [21.26, 51.55]	53.00 [29.33, 120.33]	Inf [10.24, Inf]	28.43 [20.53, 48.08]	Inf [17.02, Inf]	Inf [18.89, Inf]
**LR-**	0.30 [0.27, 0.32]	0.63 [0.61, 0.65]	0.56 [0.53, 0.58]	0.61 [0.58, 0.63]	0.80 [0.78, 0.81]	0.77 [0.75, 0.79]

1 The age stratification included infants (<1 year), children (1–11 years), and adolescents (≥12 years).
